# Structural Properties of Polyphenols Causing Cell Cycle Arrest at G1 Phase in HCT116 Human Colorectal Cancer Cell Lines

**DOI:** 10.3390/ijms140816970

**Published:** 2013-08-19

**Authors:** Soon Young Shin, Hyuk Yoon, Seunghyun Ahn, Dong-Wook Kim, Dong-Ho Bae, Dongsoo Koh, Young Han Lee, Yoongho Lim

**Affiliations:** 1 Department of Biological Sciences, Konkuk University, Seoul 143-701, Korea; E-Mails: shinsy@konkuk.ac.kr (S.Y.S.); yhlee58@konkuk.ac.kr (Y.H.L.); 2 Division of Bioscience and Biotechnology, BMIC, Konkuk University, Seoul 143-701, Korea; E-Mails: deckerglass0511@gmail.com (H.Y.); donghoya@konkuk.ac.kr (D.-H.B.); 3 Department of Applied Chemistry, Dongduk Women’s University, Seoul 136-714, Korea; E-Mails: mistahn321@naver.com (S.A.); dskoh@dongduk.ac.kr (D.K.); 4 National Institute of Animal Science, Rural Development Administration, Suwon 441-706, Korea; E-Mail: poultry98@korea.kr

**Keywords:** polyphenols, flavonoids, colorectal cancer, cell cycle, QSAR

## Abstract

Plant-derived polyphenols are being tested as chemopreventive agents; some polyphenols arrest the cell cycle at G1 phase, whereas others inhibit cell cycle proliferation at G2/M phase. Therefore, polyphenols have been proposed to inhibit cell cycle progression at different phases via distinct mechanisms. Indeed, our previous studies showed that small structural differences in polyphenols cause large differences in their biological activities; however, the details of the structural properties causing G1 cell cycle arrest remain unknown. In this study, we prepared 27 polyphenols, including eight different scaffolds, to gain insight into the structural conditions that arrest the cell cycle at G1 phase in a quantitative structure–activity relationship study. We used cell cycle profiles to determine the biophores responsible for G1 cell cycle arrest and believe that the biophores identified in this study will help design polyphenols that cause G1 cell cycle arrest.

## 1. Introduction

Colorectal cancer might be prevented by chemopreventive agents, and plant-derived polyphenols are being tested as such. Since the cancer cell cycle is faster than the normal cell cycle, cell cycle arrest influences cancer cells more than normal cells. The cell cycle can be broken down into G0/G1, S, G2, and M phases, and some polyphenols arrest the cell cycle at G1 phase, whereas others inhibit cell cycle proliferation at G2/M phase. For example, the green tea polyphenol epigallocatechin gallate (EGCG), the black tea polyphenols theaflavin and thearubigin, and the olive leaf polyphenols, hydroxytyrosol and oleuropein arrest the cell cycle at G1 phase [1–3]. In comparison, the rosemary polyphenols, carnosol and carnosic acid arrest the cell cycle at G2/M phase [4]. All of these compounds are plant-derived polyphenols.

Cell cycle progression is regulated by cyclin-dependent kinases (CDKs): CDK2/4 controls G1 phase progression, CDK2 controls S phase progression, and CDK1 controls G2/M phase progression. CDK activation requires cyclins, which are responsible for binding with CDK substrates and targeting the CDK to a specific subcellular location. G1 cell cycle progression is regulated by D-type cyclins (cyclin D). CDKN1A, also known as p21, CIP1, or WAF1, directly inhibits the activity of CDK–cyclin complexes and functions as an endogenous negative regulator of cell cycle progression [5]. Studies have demonstrated that EGCG inhibits CDK activity via induction of the CDK inhibitor CDKN1A/p21 (hereafter p21) and p27 expression [6], while resveratrol induces cell cycle arrest at the S-G2 phase transition via the overexpression of cyclins A and E without p21 expression in HL60 leukemia cells [7]. Dietary polyphenols, including anthocyanins, genistein, and sylimarin, also induce G1 cell cycle arrest by upregulating p21 expression [8]. We also found that the polyphenol, curcumin induces G1 cell cycle arrest by inducing p21 in U87MG glioma cells [9]. Consequently, polyphenols have been suggested to inhibit cell cycle progression at different phases via distinct mechanisms. Indeed, our previous studies showed that small structural differences in polyphenols cause large differences in their biological activities [10–12]. However, the detailed structural properties responsible for G1 cell cycle arrest remain unknown.

In this quantitative structure–activity relationship (QSAR) study, we prepared 27 polyphenols, including eight different scaffolds, to gain insight into the structural conditions that arrest cell cycle at G1 phase. Given that the altered expression or activities of many cell cycle regulatory molecules, including CDK inhibitors (such as p21, INK4 family, and p27) and cyclin D (D1, D2 and D3), could be involved in the dysregulation of G1 cell cycle progression, we used cell cycle profiles to determine the biophores responsible for G1 cell cycle arrest. We believe that the biophores identified in this research will help us to design polyphenols causing G1 cell cycle arrest.

## 2. Results and Discussion

The compounds examined in this study are listed in Table 1. They include three phenyliminophenols (**1**–**3**), two phenylacetamides (**4** and **5**), four phenylbenzochromenones (**6**–**9**), three benzoflavanones (**10**–**12**), seven chromenylphenylpropenones (**13**–**19**), three bischromenylpropenones (**20**–**22**), four chromenes (**23**–**26**), and one phenylchromenylpropenone (**27**).

**Figure 1:**
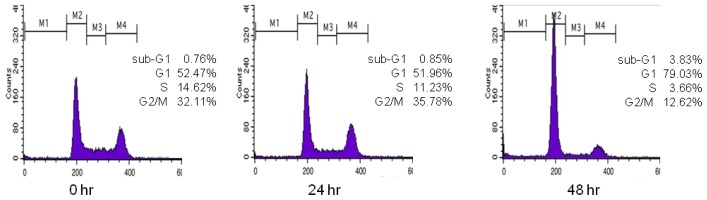
The DNA histogram of polyphenol **9** (MPBC) obtained using flow cytometry. Regions M1–M4 indicate the sub-G1, G1, S, and G2/M phases, respectively.

Cell cycle profiles were analyzed using flow cytometry. To quantify the relationships among the structures of polyphenols **1**–**27** and their effects on cell cycle arrest at G1 phase, a three-dimensional (3D) QSAR study was performed using comparative molecular field analysis (CoMFA) and comparative molecular similarity indices analysis (CoMSIA). For the QSAR calculations, the biological activity was determined as follows: Figure 1 shows the DNA histogram of polyphenol **9** obtained by flow cytometry. Regions M1–M4 indicate the sub-G1, G1, S, and G2/M phases, respectively. When a compound arrests the cell cycle at G1 phase, the percentage of cells at G1 phase should increase compared with G2/M phase. Initially, the percentage of cells in G1 phase in the histogram is 52.47%; after 24 h, it is 51.96%; and after 48 h, it becomes 79.03%. Likewise, the initial percentage of cells in G2/M phase in the histogram is 32.11%; after 24 h, it is 35.78%; and after 48 h, it becomes 12.62%. This indicates that the polyphenol **9** treatment arrested the cell cycle at G1 phase. Therefore, the ratio of the percentage of cells in G1 phase to that in G2/M phase gives information about the inhibitory effect of the test compound on cell cycle progression in G1 phase. The ratios for the 27 polyphenols tested here were obtained in this manner (Table 1). Since the logarithmic scale of these values had a better distribution than the raw data, the logarithmic values were used as the biological data for the QSAR calculations (Table 1). Polyphenol **9** or 2-(3-methoxyphenyl)-2*H-*benzo[ *h*]chromen-4(3*H*)-one (MPBC), which best inhibited the G1 phase cell cycle progression, was used as a template.

Among the models generated from the CoMFA, the model with the best cross-validated correlation coefficient (*q*^2^ = 0.754) was chosen for further analysis. Partial least square (PLS) analysis was used to establish a linear relationship between the biological activity and resulting field matrix of the 27 polyphenols. The cross-validated analysis was performed using the leave-one-out (LOO) method. The final non-cross-validated (*r*^2^ = 0.999) analysis was performed using the optimal number of components (*n* = 6) obtained from the LOO method. In the PLS analysis, the standard error of the estimate was 0.008 and the F-value was 2068.154. To evaluate the CoMFA model, the activities of the compounds in the training set were predicted and compared to the experimental data (Table S1). The residuals between the experimental and predicted values for the training set ranged from 0.03% to 0.67%. To validate the QSAR model, five compounds (**5**, **10**, **16**, **22**, and **23**) were selected randomly as a test set. Their residuals ranged from 4.61% to 14.09%. Therefore, the resulting CoMFA model is reliable. The experimental data are plotted against the predicted values (Figure S1). While CoMFA provides information only on the steric and electrostatic effects, CoMSIA also gives information on the hydrophobic, H-bond donor, and acceptor effects. From among several CoMSIA models, the model with the best cross-validated value (*q*^2^ = 0.673) was selected. The correlation coefficient (*r*^2^) was 0.993. Six components were in the PLS statistical parameters, the standard error of the estimate was 0.020, and the F-value was 337.846. Like CoMFA, to evaluate the CoMSIA model, the activities for the training and test sets were predicted and compared to the experimental data (Table S1). The experimental data are plotted against the predicted value (Figure S2). In the training set, the residuals between the experimental and predicted values ranged from 0 to 1.74%; in the test set, the range was 4.35%–13.10%. Therefore, the CoMSIA model is reliable.

**Figure 2:**
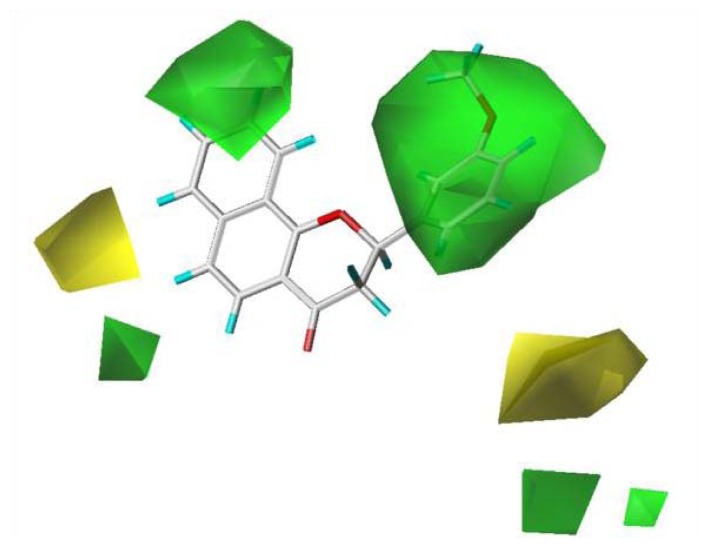
CoMFA contour map generated using the program SYBYL 7.3. The steric and electrostatic field descriptors contributed 44.7% and 55.3%, respectively. Regarding the steric field, the region favoring steric bulk contributed 19% and that disfavoring bulk contributed 81%. Similarly, a contour map for the electrostatic field was generated, and the regions favoring electropositive and negative groups contributed 14% and 86%, respectively. MPBC had the best inhibitory effect and is embedded in the contour map.

To visualize the relationships among the structures of the 27 polyphenols and their inhibition of the G1 phase of cell cycle progression, CoMFA contour maps were generated using the program SYBYL 7.3. The steric and electrostatic field descriptors contributed 44.7% and 55.3%, respectively. Regarding the steric field, the region favoring steric bulk contributed 19% and the region not favoring bulk contributed 81%. MPBC had the greatest inhibitory effect and is embedded in the contour map (Figure 2). Similarly, a contour map for the electrostatic field was generated, in which the regions favoring electropositive and electronegative groups contributed 14% and 86%, respectively. The bulky substituents at C-2 of the chromene group contribute positively to the activity, as polyphenols **6**–**12** show greater activity than other polyphenols without bulky substituents at the C-2 of chromene. The 7,8-naphtho group had better activity than the 5,6-naphtho group, as the activities of polyphenols **6**–**9** were better than those of **10**–**12**. The chromenyl group at the propenone of **13**–**22** decreased the activity. The electronegative group at C-4 of the chromene moiety increased the activity.

**Figure 3:**
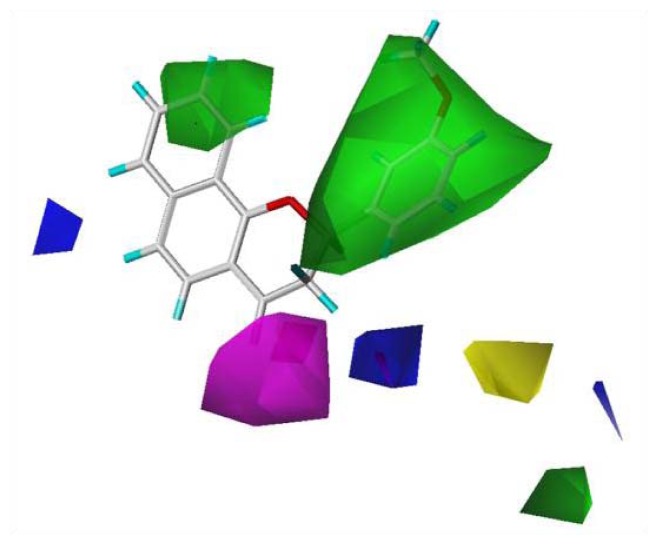
CoMSIA contour map generated using the program SYBYL 7.3. The steric, electrostatic, and H-bond acceptor field descriptors contributed 30.8%, 51.0%, and 18.2%, respectively. Regarding the steric field, the region favoring steric bulk contributed 12% and that disfavoring bulk contributed 88%; in the electrostatic field, the region favoring electropositive groups contributed 90% and that favoring electronegative groups contributed 10%. For the H-bond acceptor field, the region favoring H-bond acceptors contributed 79% and that disfavoring H-bond acceptors contributed 21%.

Using the same method as with the CoMFA, a CoMSIA contour map was generated. In addition to information about the steric and electrostatic fields, CoMSIA provides information on the hydrophobic, H-bond donor, and acceptor fields. In this experiment, the CoMSIA model selected only the steric, electrostatic, and H-bond acceptor field descriptors, which contributed 30.8%, 51.0%, and 18.2%, respectively. The CoMSIA contour maps show where more bulk, less bulk, electronegative groups, electropositive groups, and H-bond acceptors were favored, and where H-bond acceptors were disfavored (Figure 3). Regarding the steric field, the region favoring steric bulk contributed 12% and the region disfavoring bulk contributed 88%. Regarding the electrostatic field, the region favoring electropositive groups contributed 90% and that favoring electronegative groups contributed 10%. For the H-bond acceptor field, the region favoring H-bond acceptors contributed 79% and that disfavoring H-bond acceptors contributed 21%. The CoMSIA and CoMFA steric and electrostatic field contour maps showed similar trends because steric field descriptors contributed less than electrostatic field descriptors in both CoMSIA and CoMFA; therefore, the contour maps for the H-bond acceptor fields were analyzed. While the H-bond acceptor at the C-4 of chromene moiety contributed to the activity, the H-bond acceptor of the substituents at the C-3 position of the chromene moiety did not.

**Figure 4:**
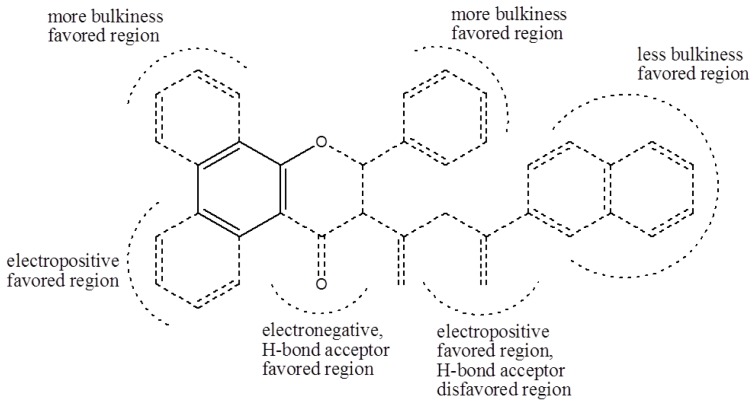
The structural conditions resulting in greater inhibition of cell cycle progression in G1 phase in HCT116 human colorectal cancer cells.

Based on the analysis of the CoMFA and CoMSIA contour maps, biophores that markedly inhibit cell cycle progression at G1 phase can be described, as shown in Figure 4. Solid lines indicate the benzyl moiety used to align the polyphenols and dotted lines indicate substituents contained in some of the polyphenols tested.

**Figure 5:**
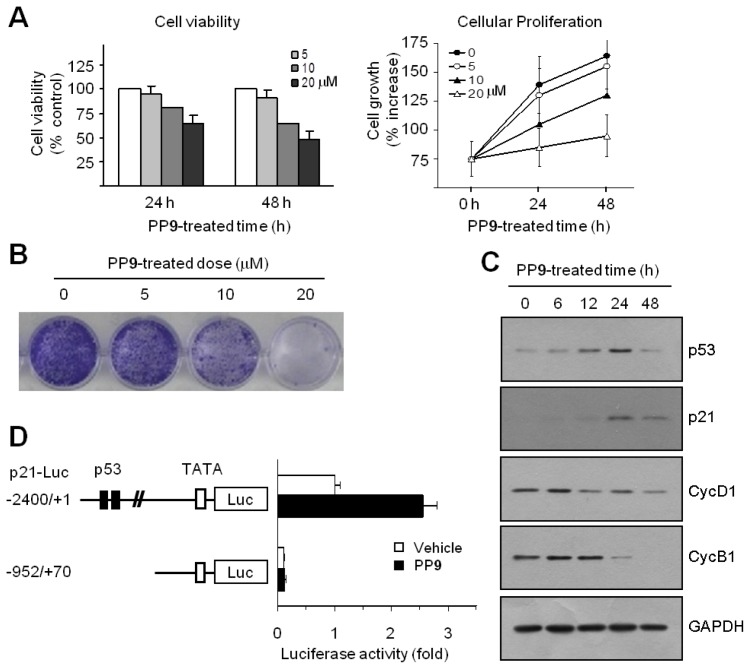
(**A**) Treatment with MPBC significantly reduced cell viability (**left**) and cellular proliferation (**right**) in a dose- and time-dependent manner; (**B**) Treatment with MPBC for 7 days resulted in a dose-dependent loss of the ability of individual cells to proliferate into viable colonies; (**C**) Western blot analysis showed that the amount of p53 protein increased within 6 h following MPBC treatment, reached a peak around 24 h, and then dropped by 48 h; (**D**) Treatment with MPBC resulted in the stimulation of the −2400/+1 construct, but not the −952/+70 construct.

The inhibition of cell cycle progression is often associated with an antiproliferative mechanism. To assess the antiproliferative activity of polyphenol **9** (2-(3-methoxyphenyl)-2*H*-benzo[*h*]chromen-4(3*H*)-one, MPBC), exponentially growing HCT116 cells were exposed to various concentrations of MPBC for 24 or 48 h, and cell viability was measured using a Cell Counting Kit-8 Assay kit. A decrease in cell viability was observed in cells treated with MPBC in a dose- and time-dependent manner (Figure 5A, left graph). Cellular proliferation rate was measured using a Cell Proliferation Assay Kit based on the incorporation of bromo-deoxyUridine (BrdU) during DNA synthesis. Treatment with MPBC also reduced cell proliferation rate in a dose- and time-dependent manner compared with untreated control cells (Figure 5A, right graph). The clonogenic survival assay is based on the ability of a single tumor cell to grow into a viable colony and considered to be a reliable test for predicting the effectiveness of anticancer agents. To assess the long-term efficacy of MPBC at inhibiting tumor cell growth, the clonogenic survival assay was performed. Treatment with MPBC for 7 days resulted in a dose-dependent loss of the ability of individual cells to proliferate into viable colonies (Figure 5B). The CDK inhibitor p21 is regarded as the most potent cell cycle regulator involved in blocking G1 cell cycle progression. p21 expression is controlled in a tumor suppressor p53-dependent and p53-independent manner. To investigate the molecular mechanism underlying MPBC-induced G1 cell cycle arrest, the expression levels of p53 and p21 were examined. Western blot analysis showed that the amount of p53 protein increased within 6 h following MPBC treatment, reached a peak at around 24 h, and dropped thereafter by 48 h (Figure 5C). The amount of p21 protein began to increase after 6 h, with a maximum at approximately 24 h, after which the level dropped considerably by 48 h, but still remained high compared to the basal level. In contrast, the levels of cyclins D1 and B1 both decreased within 24 h, suggesting that the MPBC-induced accumulation of p53 and p21 proteins induces G1 cell cycle arrest, thereby altering the expression of G1 and G2/M regulatory cyclins. To establish whether MPBC-induced p21 expression is mediated via p53-dependent transcriptional activation, the p21 promoter reporter assay was performed. HCT116 cells were transfected with p21-Luc(−2400/+1), a p21 promoter-reporter construct with 2.4 kb of the 5′-flanking sequence, or with p21-Luc(−952/+70), a deletion construct lacking the p53 consensus binding sites. Then, the luciferase activity was measured. Treatment with MPBC resulted in the stimulation of the −2400/+1 construct, but not the −952/+70 construct (Figure 5D). These data suggest that MPBC increases p21 expression via p53-dependent transcriptional activation of the *p21* gene.

**Figure 6:**
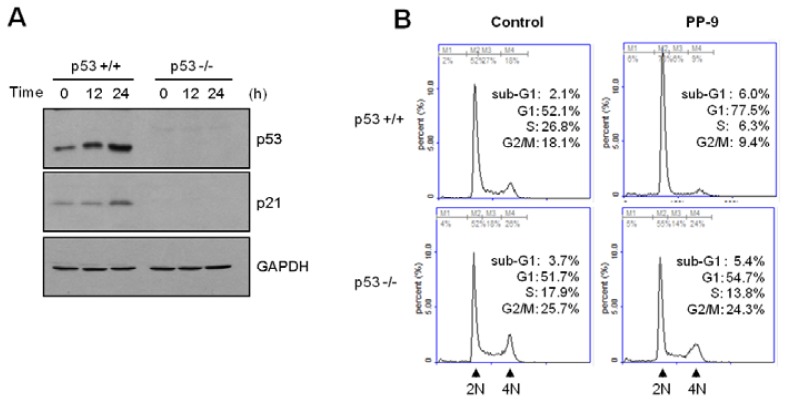
(**A**) The expression of both p53 and p21 were time-dependently increased following MPBC exposure of the wild-type HCT116 cells (p53^+/+^), but not p53-null HCT116 cells (p53^−/−^); (**B**) MPBC-induced G1 cell cycle arrest was impaired in p53-null HCT116 cell.

To corroborate the role of p53 in MPBC-induced p21 expression, we used p53-null HCT116 cells [13]. As shown in Figure 6A, both p53 and p21 expression were time-dependently increased following the exposure of wild-type HCT116 cells (p53^+/+^), but not p53-null HCT116 cells (p53^−/−^), to MPBC. Furthermore, the MPBC-induced G1 cell cycle arrest was impaired in p53-null HCT116 cells (Figure 6B). These results indicate that MPBC-induced G1 cell cycle arrest is mediated via the upregulation of p21 following the transcriptional activation of p53 in HCT116 cells.

## 3. Experimental Section

All of the polyphenols examined in this study except **6** and **9** were synthesized using reported methods [14–27]. The synthetic procedures for novel polyphenols **6** and **9**, and nuclear magnetic resonance (NMR) and mass spectroscopy (MS) data to used identify them are described here. The synthesis procedures are shown in Scheme S1. Melting points (m.p.) were determined on a Fisher–Johns melting apparatus (Fisher Scientific, Lafayette, CO, USA) and were uncorrected. All NMR experiments were performed on an Avance 400 spectrometer system (9.4 T; Bruker, Karlsruhe, Germany) at 298 K. The synthetic compounds were dissolved in deuterated dimethyl sulfoxide (DMSO-d_6_). The detailed experimental methods followed reported methods [23]. All mass spectra were collected on a high-resolution electron impact ionization mass spectrometer (HREIMS; JMS700, JEOL, Tokyo, Japan) with the help of the Korea Basic Science Institute at Daegu, Korea. Column chromatography purification was performed on Silica gel 60 (70–230 mesh, Merck, Whitehouse Station, NJ, USA) [28].

Synthesis of 2-(2,3-dimethoxyphenyl)-2*H*-benzo[*h*]chromen-4(3*H*)-one (**6**). 1-Hydroxy-2-acetonaphthone (558 mg, 3 mmol) and 2,3-dimethoxybenzaldehyde (498 mg, 3 mmol) were dissolved in 40 mL of ethanol and the temperature was adjusted to 3–4 °C in an ice bath. To the cooled reaction mixture was added 2 mL of 50% aqueous KOH solution, and the reaction mixture was stirred at room temperature for 24 h. The reaction mixture was poured into iced water (60 mL) and acidified with 6 N HCl solution to give a solid. The resulting precipitate was filtered and washed with cold ethanol to give a pure chalcone intermediate, which was used for the next reaction. To a solution of chalcone (668 mg, 2 mmol) in 10 mL of dimethyl formamide (DMF) was added a catalytic amount of 6 N HCl and the mixture was refluxed for 20 h. After cooling, the resulting solution was poured into iced water (200 mL) to give a crude solid. The mixture was extracted with CH_2_Cl_2_ (20 mL × 3). The combined organic layers were dried over anhydrous MgSO_4_ and the solvent was evaporated *in vacuo*. After flash column chromatography (ethyl acetate:*n*-hexane = 1:20), pure flavanone **6** was obtained in 53% yield; m.p. 112–114 °C. ^1^H NMR (400 MHz, DMSO-d_6_) δ 8.20 (d, 1H, H-8a, *J* = 8.3 Hz), 7.95 (d, 1H, H-7a, *J* = 8.1 Hz), 7.80 (d, 1H, H-5, *J* = 8.7 Hz), 7.70 (ddd, 1H, H-7b, *J* = 1.1, 7.0, 8.1 Hz), 7.58 (m, 1H, H-8b), 7.56 (d, 1H, H-6, *J* = 8.7 Hz), 7.30 (dd, 1H, H-6′, *J* = 1.5, 8.0 Hz), 7.21 (dd, 1H, H-5′, *J* = 8.0, 8.0 Hz), 7.15 (dd, 1H, H-4′, *J* = 1.5, 8.0 Hz), 6.05 (dd, 1H, H-2, *J* = 2.9, 13.7 Hz), 3.86 (s, 3H, 3′-OCH_3_), 3.81 (s, 3H, 2′-OCH_3_), 3.36 (dd, 1H, H-3, *J* = 13.7, 16.8 Hz), 2.83 (dd, 1H, H-3, *J* = 2.9, 16.8 Hz); ^13^C NMR (100 MHz, DMSO-d_6_) δ 191.2 (C-4), 159.2 (C-9), 152.4 (C-3′), 146.1 (C-2′), 136.9 (C-7), 131.9 (C-1′), 129.8 (C-7b), 128.0 (C-7a), 126.7 (C-8b), 124.4 (C-5′), 124.2 (C-8), 123.0 (C-8a), 121.3 (C-5), 120.9 (C-6), 118.7 (C-6′), 115.0 (C-10), 113.4 (C-4′), 75.4 (C-2), 60.7 (2′-OCH_3_), 55.8 (3′-OCH_3_); HREIMS (*m*/*z*): Calcd. for C_21_H_18_O_4_ (M^+^): 334.1205; found 334.1201.

Synthesis of 2-(3-methoxyphenyl)-2*H*-benzo[*h*]chromen-4(3*H*)-one (**9**). The same procedures were used for the synthesis of polyphenol **9**, but starting from 1-hydroxy-2-acetonaphthone and 3-methoxybenzaldehyde. The compound was obtained in 47% yield; m.p. 126–128 °C. ^1^H NMR (400 MHz, DMSO-d_6_) δ 8.26 (d, 1H, H-8a, *J* = 7.9 Hz), 7.95 (d, 1H, H-7a, *J* = 8.1 Hz), 7.78 (d, 1H, H-5, *J* = 8.7 Hz), 7.71 (ddd, 1H, H-7b, *J* = 0.8, 6.9, 8.1 Hz), 7.61 (ddd, 1H, H-8b, *J* = 0.8, 6.9, 7.9 Hz), 7.55 (d, 1H, H-6, *J* = 8.7 Hz), 7.39 (dd, 1H, H-5′, *J* = 8.1, 8.1 Hz), 7.21 (m, 1H, H-2), 7.21 (m, 1H, H-6′), 6.98 (dd, 1H, H-4′, *J* = 2.4, 8.1 Hz), 5.88 (dd, 1H, H-2, *J* = 3.1, 13.0), 3.80 (s, 3H, 3′-OCH_3_), 3.34 (dd, 1H, H-3, *J* = 13.0, 16.7 Hz), 2.98 (dd, 1H, H-3, *J* = 3.1, 16.7 Hz); ^13^C NMR (100 MHz, DMSO-d_6_) δ 191.0 (C-4), 159.4 (C-3′), 158.9 (C-9), 140.4 (C-1′), 136.9 (C-7), 129.8 (C-7b), 129.8 (C-5′), 128.0 (C-7a), 126.7 (C-8b), 124.2 (C-8), 123.0 (C-8a), 121.2 (C-5), 120.9 (C-6), 118.5 (C-6′), 115.2 (C-10), 113.8 (C-4′), 112.2 (C-2′), 79.5 (C-2), 55.1 (3′-OCH_3_), 42.8 (C-3); HREIMS (*m*/*z*): Calcd. for C_20_H_16_O_3_ (M^+^): 304.1099; found 304.1097.

Cell cycle profiles were analyzed using flow cytometry. HCT116 cells were treated with either vehicle (dimethyl sulfoxide, DMSO) or 20 μM polyphenol compound for 24 or 48 h, fixed in 70% ethanol, washed twice with phosphate-buffered saline, and then stained with 50 μg/mL propidium iodide, as described previously [29]. Cellular DNA was measured using a NucleoCounter NC-3000 (ChemoMetec, Allerød, Denmark). The four distinct phases of the cell cycle were based on the cellular DNA content: G1 (gap 1; diploid), S (DNA synthesis; between diploid and tetraploid), G2 (gap 2; tetraploid), and M (mitosis; tetraploid).

To quantify the relationships among the structures of polyphenols **1**–**27** and their effects on cell cycle arrest at G1 phase, a three-dimensional (3D) QSAR study was performed using comparative molecular field analysis (CoMFA) and comparative molecular similarity indices analysis (CoMSIA). All calculations were carried out on an Intel Core 2 Quad Q6600 (2.4 GHz) Linux PC with the molecular modeling package SYBYL 7.3 (Tripose, St. Louis, MO, USA) [11]. The 3D structures of synthetic polyphenols **10**–**19** were constructed based on the X-ray crystallographic structure of 2′,3,5-trimethoxychalcone, which was determined by the authors (Figure S3A) [30]. The constructed polyphenols **10**–**19** were subjected to energy minimization using the molecular mechanics algorithms provided by SYBYL 7.3. A conformational search was performed using the grid search method of SYBYL, in which the selected bond was rotated from 0 to 360° in 15° increments, and the conformation was minimized using the Tripos force field and Gästeiger–Hückel charge. Minimization was stopped on convergence of the total energy (0.05 kcal/mol·Å). Likewise, the 3D structures of polyphenols **6**–**9** and **20**–**27** were constructed based on the X-ray crystallographic structures of 2′-hydroxy-3′,4′-naphtho-3,5-dimethoxychalcone and 8-methoxy-2*H*-chromene-3-carbaldehyde (**23**), respectively, published by the authors (Figure S3B,C) [31,32]. The 3D structures of polyphenols **1**–**5** were determined using molecular modeling. For the QSAR calculations, since the compounds in the data set are not homogeneous, heavy atoms contained in the benzyl moiety were used for alignment when a consistent superposition was observed. The alignment procedure was performed using DATABASE Alignment module in the program SYBYL. The aligned structures are shown in Figure S4.

The 27 polyphenols in Table 1 were divided randomly into a training set of 22 compounds and a test set of five compounds (**5**, **10**, **16**, **22**, and **23**). The training set was used to create QSAR models, and the test set was used to validate the models. The test set was analyzed using hierarchical clustering [33]. The compounds chosen for the test set belonged to separate structural groups (Figure S5). Therefore, the test set can be used to validate whether the QSAR models in this experiment are reliable. The QSAR calculations were performed using CoMFA and CoMSIA and the experimental procedures followed reported methods [11].

For the cell growth inhibition assay, HCT116 human colon cancer cells were obtained from the American Type Culture Collection (Manassas, VA, USA) and maintained in Dulbecco’s modified Eagle’s medium (DMEM; Life Technologies, Seoul, Korea) supplemented with 10% fetal bovine serum (FBS; HyClone, Logan, UT, USA). The HCT116 cells (2 × 10^3^ cells/sample) were seeded into 96-well plates and treated with MPBC at increasing concentrations (0, 5, 10, and 20 μM) for 24 or 48 h. The inhibitory effect of MPBC on cell viability was determined using the Cell Counting Kit-8™ (Dojindo Molecular Technologies, Gaithersburg, MD) with the water-soluble tetrazolium salt WST-8 (2-(2-methoxy-4-nitrophenyl)-3-(4-nitrophenyl)-5-(2,4-disulfophenyl)-2*H*-tetrazolium, monosodium salt) as the substrate, according to the manufacturer’s instructions [34]. The inhibitory effect of MPBC on cellular proliferation was measured using a Cell Proliferation Assay Kit (Cell Signaling Technology, Danvers, MA, USA), according to the manufacturer’s instructions. For the clonogenic survival assay, HCT116 cells were counted and plated onto 24-well tissue culture plates (BD Falcon™; Becton Dickson Immunocytometry System, Franklin Lakes, NJ, USA; 5 × 10^3^ cells/well) in DMEM supplemented with 10% FBS. After attachment, the cells were treated with MPBC for 7 days. Then, the cells were fixed with 6% glutaraldehyde and stained with 0.1% crystal violet, as described previously [35]. For the Western blot analysis, cells were lysed in a buffer containing 20 mM HEPES (4-(2-hydroxyethyl)-1-piperazineethanesulfonic acid, pH 7.2), 1% Triton X-100, 10% glycerol, 150 mM NaCl, 10 μg/mL leupeptin, and 1 mM phenylmethylsulfonyl fluoride (PMSF). Western blotting was performed as described previously [34]. Signals were developed using an enhanced chemiluminescence detection system (Amersham Pharmacia Biotech, Piscataway, NJ, USA).

For the promoter reporter assay, HCT116 cells were seeded onto 12-well plates and transfected with 0.2 μg of one of two p21 promoter constructs, p21-Luc(−2400/+1) or p21-Luc(−952/+70) using LipofectAMINE 2000 (Invitrogen Life Technologies, San Diego, CA, USA). To monitor the transfection efficiency, a pRL-null plasmid (50 ng) encoding *Renilla* luciferase was included in all transfections. At 24 h post-transfection, the cells were treated with 20 μM MPBC. After 8 h, the levels of firefly and *Renilla* luciferase activity were measured sequentially from a single sample using the Dual-Glo Luciferase Assay System, according to the manufacturer’s instructions. Luminescence was measured with a luminometer (Centro LB960; Berthold Tech, Bad Wildbad, Germany).

## 4. Conclusions

The structural conditions required for marked inhibition of G1 cell cycle progression were summarized based on the CoMFA and CoMSIA. The QSAR study demonstrated that several moieties shown with dotted lines in Figure 4 are necessary for G1 cell cycle arrest of HCT116 colon cancer cells. Biological mode of action studies revealed that 2-(3-methoxyphenyl)-2*H*-benzo[*h*]chromen-4(3*H*)-one (polyphenol **9**, MPBC) induces transcription factor p53-mediated upregulation of the cell cycle inhibitor p21. Silencing the p53 protein abrogated the MPBC-induced G1 cell cycle arrest. Based on these data, we suggest that MPBC exhibits antitumor activity via the p53-mediated inhibition of cell cycle progression at G1 phase. The structural conditions producing cell cycle arrest at G1 phase determined based on QSAR have never been reported. The biophores obtained in this study might help us to design polyphenols that trigger G1 cell cycle arrest.

## Supplementary Information



## Figures and Tables

**Table 1:** Structures and names of the 27 polyphenols and the logarithmic scale of their inhibition of cell cycle progression of G1 phase in HCT116 human colorectal cancer cells. The asterisk (*) denotes the test set used to calculate the quantitative structure-activity relationships.

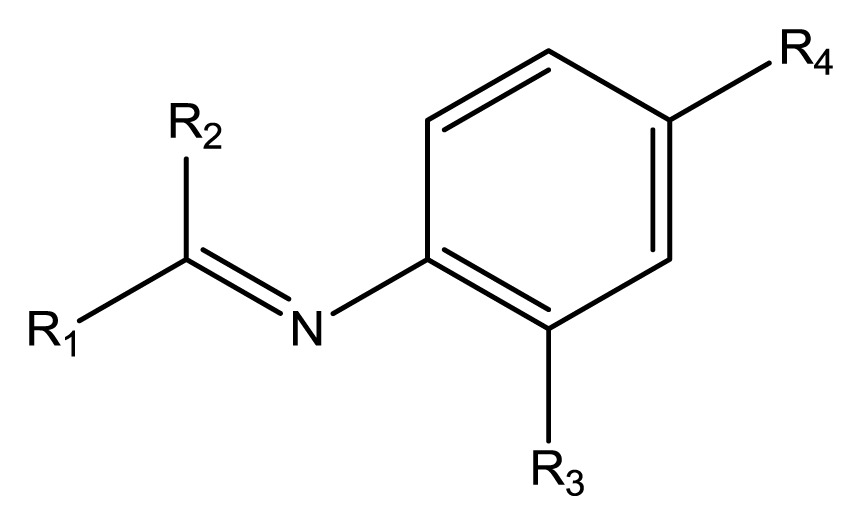							
Polyphenol	Name	R_1_	R_2_	R_3_	R_4_	Activity	log(activity)
**1**	(*E*)-2-methoxy-6-((2-methoxyphenylimino)methyl)phenol	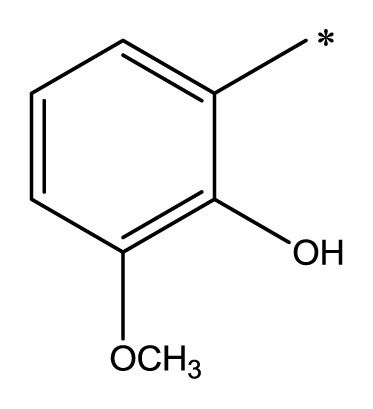	H	OCH_3_	H	53.88	1.73
**2**	(*E*)-2-((4-methoxyphenylimino)methyl)phenol	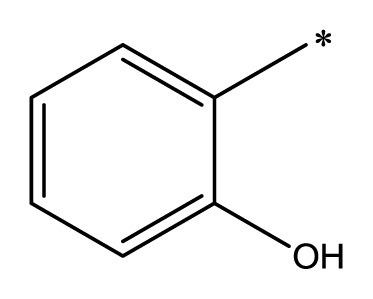	H	H	OCH_3_	48.00	1.68
**3**	(*E*)-2-(1-(4-methoxyphenylimino)ethyl)naphthalen-1-ol	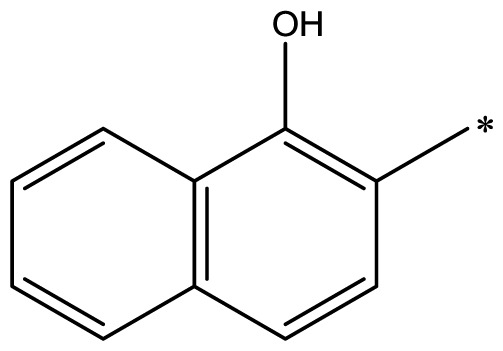	CH_3_	H	OCH_3_	47.18	1.67

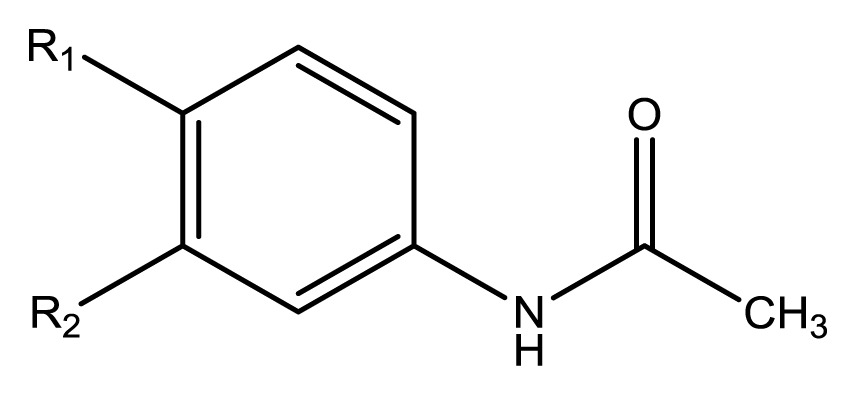					
Polyphenol	Name	R_1_	R_2_	Activity	log(activity)
**4**	*N*-(3-methoxyphenyl)acetamide	H	OCH_3_	49.86	1.70
5*	*N*-(4-methoxyphenyl)acetamide	OCH_3_	H	45.13	1.65

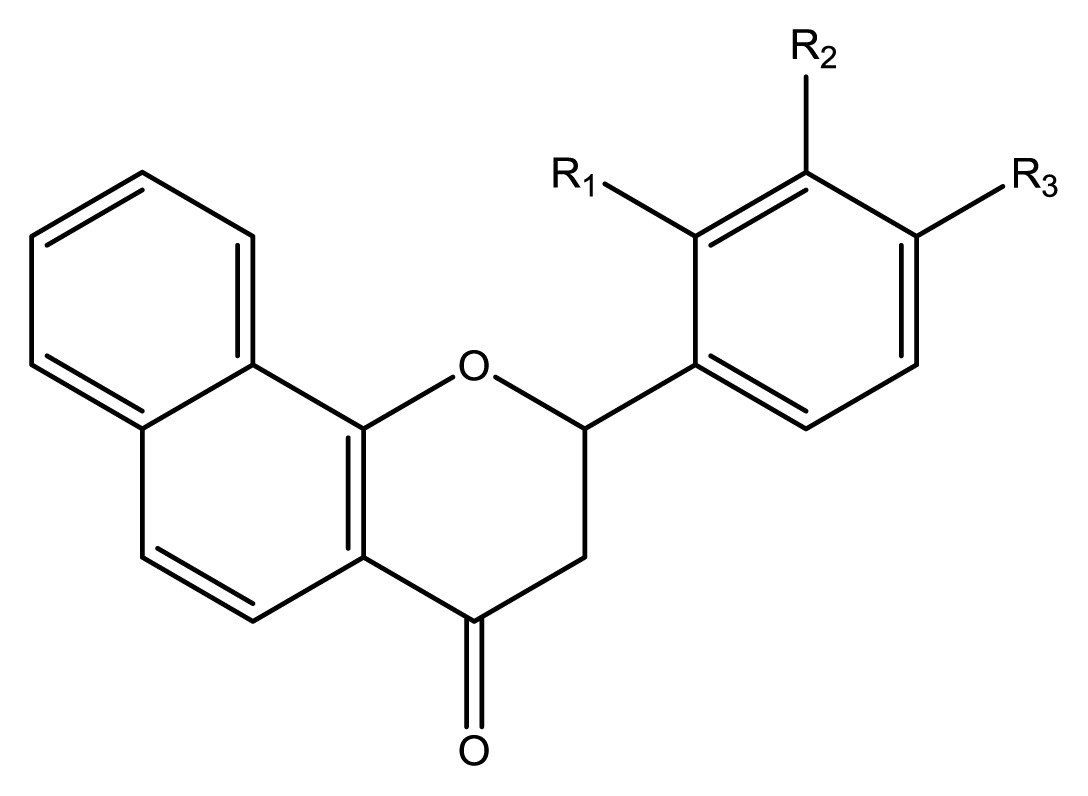						
Polyphenol	Name	R_1_	R_2_	R_3_	Activity	log(activity)
**6**	2-(2,3-dimethoxyphenyl)-2*H*benzo[*h*]chromen-4(3*H*)-one	OCH_3_	OCH_3_	H	38.72	1.59

**7**	2-(2-methoxyphenyl)-2*H*-benzo[*h*]chromen-4(3*H*)-one	OCH_3_	H	H	41.47	1.62

**8**	2-(4-methoxyphenyl)-2*H*-benzo[*h*]chromen-4(3*H*)-one	H	H	OCH_3_	36.43	1.56
**9**	2-(3-methoxyphenyl)-2*H*-benzo[*h*]chromen-4(3*H*)-one	H	OCH_3_	H	36.27	1.56

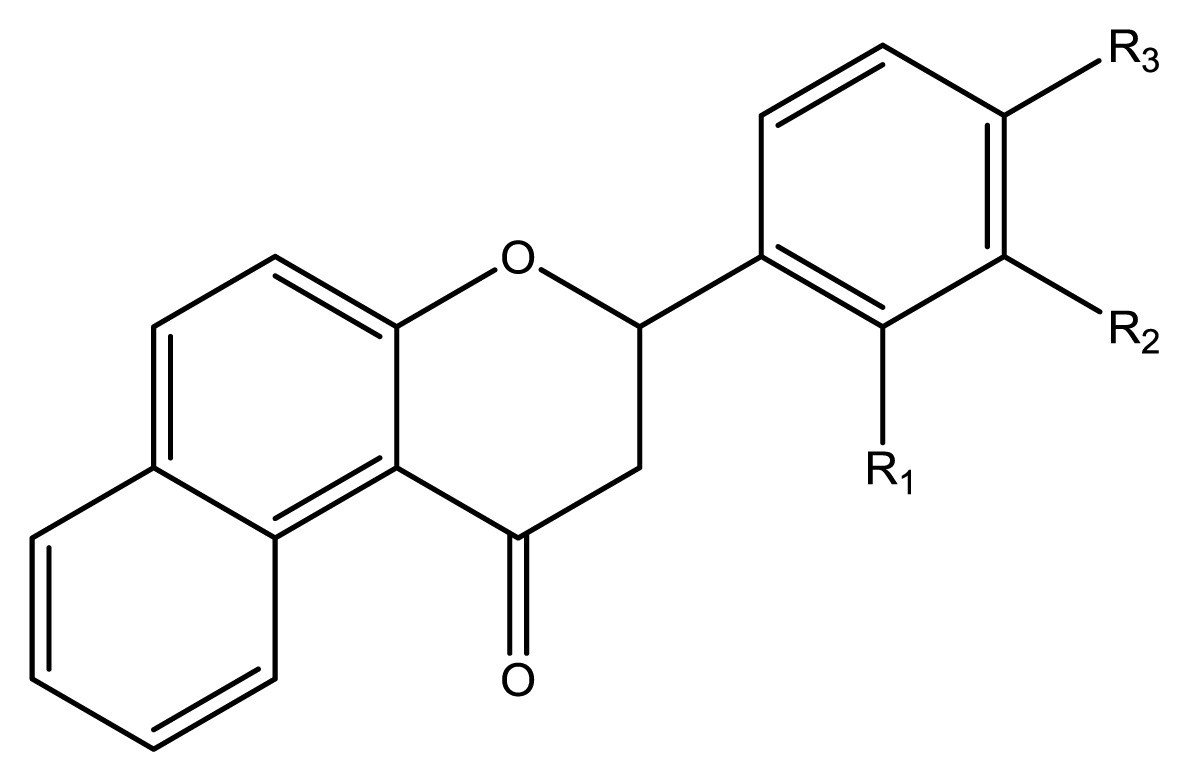						
Polyphenol	Name	R_1_	R_2_	R_3_	Activity	log(activity)
10 *	3-(4-methoxyphenyl)-2,3-dihydro-1*H*benzo[*f*]chromen-1-one	H	H	OCH_3_	57.09	1.76
**11**	3-(2,3-dimethoxyphenyl)-2,3-dihydro-1*H*benzo[*f*]chromen-1-one	OCH_3_	OCH_3_	H	44.35	1.65
**12**	3-(2,4-dimethoxyphenyl)-2,3-dihydro-1*H*benzo[*f*]chromen-1-one	OCH_3_	H	OCH3	46.04	1.66

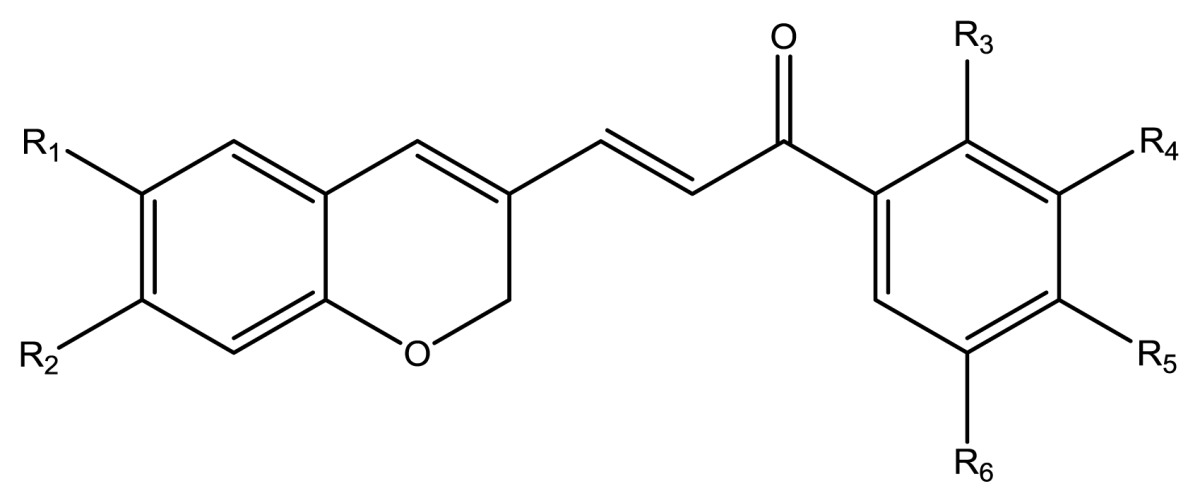									
polyphenol	Name	R_1_	R_2_	R_3_	R_4_	R_5_	R_6_	Activity	log(activity)
**13**	(*E*)-3-(6-methoxy-2*H*-chromen-3-yl)-1-(4-methoxyphenyl)prop-2-en-1-one	OCH_3_	H	H	H	OCH_3_	H	92.58	1.97
**14**	(*E*)-1-(3,5-dimethoxyphenyl)-3-(6-methoxy-2*H*-chromen-3-yl)prop-2-en-1-one	OCH_3_	H	H	OCH_3_	H	OCH_3_	117.88	2.07
**15**	(*E*)-3-(7-methoxy-2*H*-chromen-3-yl)-1-(4-methoxyphenyl)prop-2-en-1-one	H	OCH_3_	H	H	OCH_3_	H	69.88	1.84
16*	(*E*)-3-(6-methoxy-2*H*-chromen-3-yl)-1-(2-methoxyphenyl)prop-2-en-1-one	OCH_3_	H	OCH_3_	H	H	H	257.53	2.41
**17**	(*E*)-3-(6-bromo-2*H*-chromen-3-yl)-1-(3-methoxyphenyl)prop-2-en-1-one	Br	H	H	OCH_3_	H	H	61.54	1.79
**18**	(*E*)-3-(6-bromo-2*H*-chromen-3-yl)-1-(2-methoxyphenyl)prop-2-en-1-one	Br	H	OCH_3_	H	H	H	97.42	1.99
**19**	(*E*)-3-(6-bromo-2*H*-chromen-3-yl)-1-(3,5-dimethoxyphenyl)prop-2-en-1-one	Br	H	H	OCH_3_	H	OCH_3_	77.76	1.89

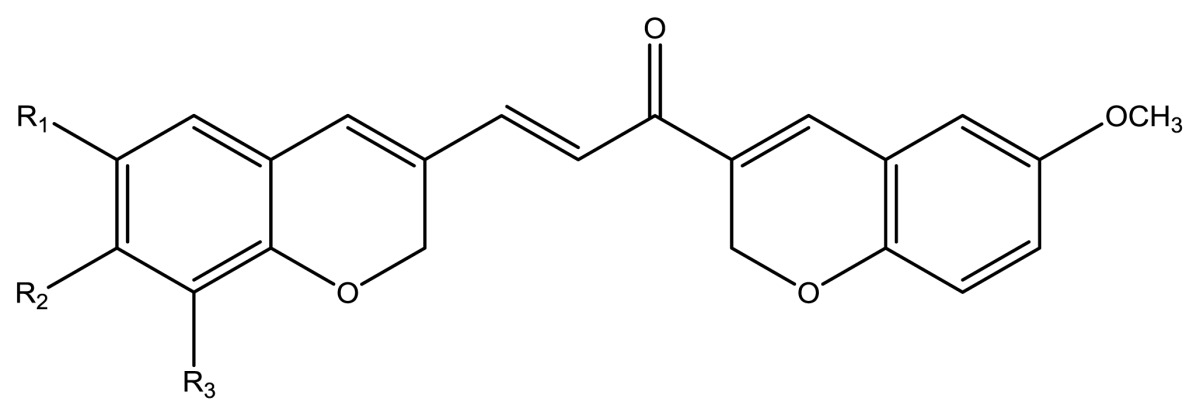						
Polyphenol	Name	R_1_	R_2_	R_3_	Activity	log(activity)
**20**	(*E*)-1,3-bis(6-methoxy-2*H*-chromen-3-yl)prop-2-en-1-one	OCH_3_	H	H	102.15	2.01
**21**	(*E*)-1-(6-methoxy-2*H*-chromen-3-yl)-3-(7-methoxy-2*H*chromen-3-yl)prop-2-en-1-one	H	OCH_3_	H	94.81	1.98
22*	(*E*)-1-(6-methoxy-2*H*-chromen-3-yl)-3-(8-methoxy-2*H*chromen-3-yl)prop-2-en-1-one	H	H	OCH_3_	101.39	2.01

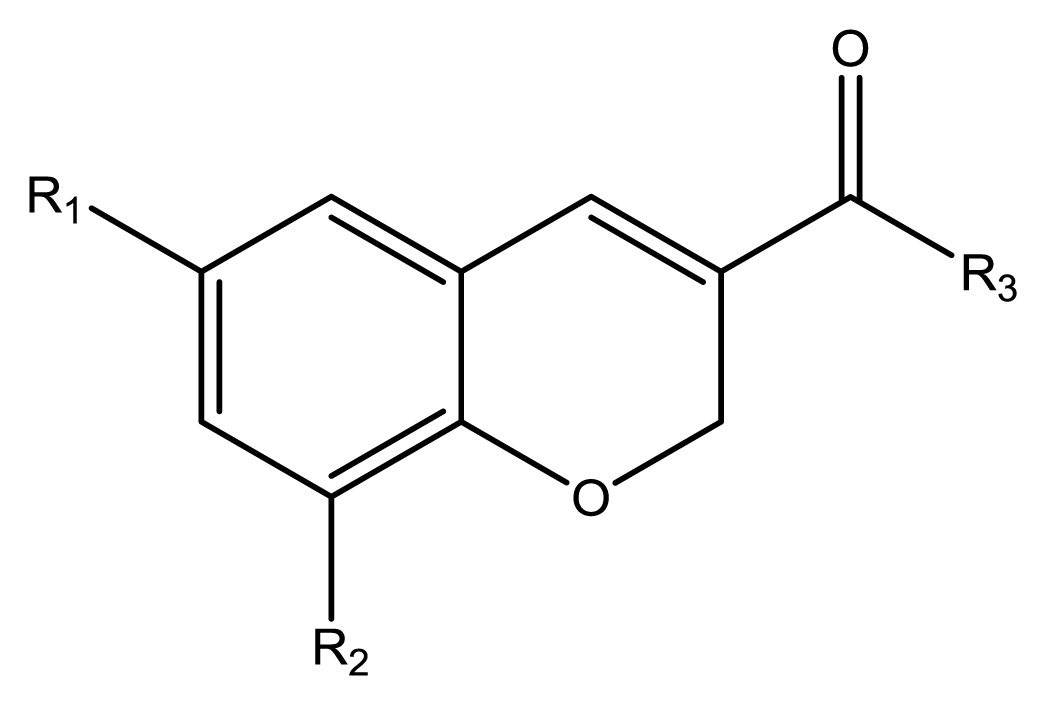						
Polyphenol	Name	R_1_	R_2_	R_3_	Activity	log(activity)
23*	8-methoxy-2*H*-chromene-3-carbaldehyde	H	OCH_3_	H	42.27	1.63
**24**	1-(8-methoxy-2*H*-chromen-3-yl)ethanone	H	OCH_3_	OCH_3_	60.46	1.78
**25**	2*H*-chromene-3-carbaldehyde	H	H	H	57.82	1.76
**26**	6-bromo-2*H*-chromene-3-carbaldehyde	Br	H	H	60.74	1.78

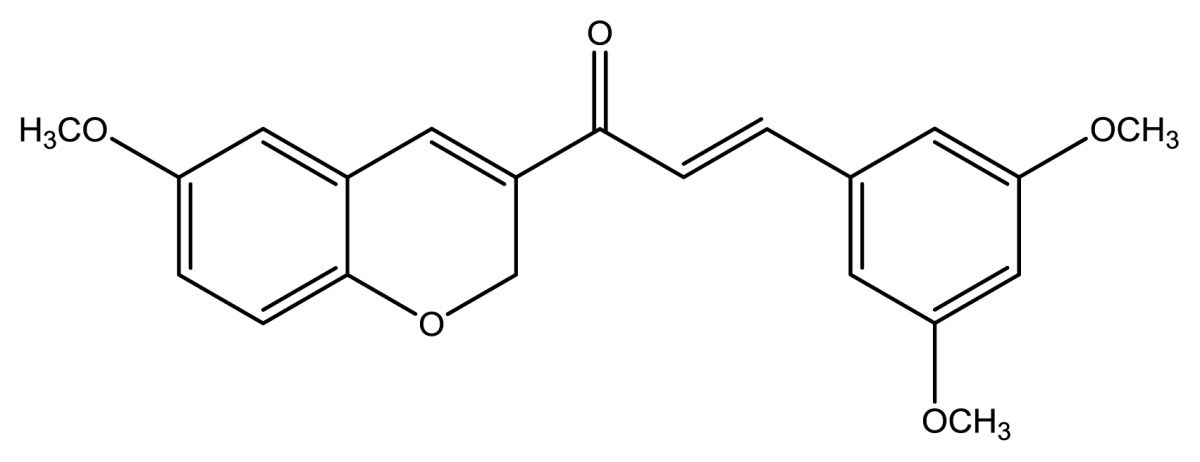			
Polyphenol	Name	Activity	log(activity)
**27**	(*E*)-3-(3,5-dimethoxyphenyl)-1-(6-methoxy-2*H*-chromen-3-yl)prop-2-en-1-one	224.94	2.35
