# Supplementary material for: Structural Properties of Polyphenols Causing Cell Cycle Arrest at G1 Phase in HCT116 Human Colorectal Cancer Cell Lines
Source: Int J Mol Sci. 2013 Aug 19;14(8):16970–85. doi: 10.3390/ijms140816970 (PMC3759946; doi:10.3390/ijms140816970)
Supplement: Supplementary file 1 [file ijms-14-16970-s001.pdf]

Supplementary Information

Table S1. Experimental and predicted values with residual values for the training set and test set. Asterisk (*) denotes the test set.

	Experimental data	CoMFA		CoMSIA	
		Predicted	Residual/%	Predicted	Residual/%
**1**	1.73	1.718	0.67	1.730	0.00
**2**	1.68	1.687	−0.41	1.697	−1.02
**3**	1.67	1.675	−0.29	1.679	−0.53
**4**	1.70	1.701	−0.03	1.670	1.74
**5 ***	1.65	1.828	−10.80	1.792	−8.62
**6**	1.59	1.585	0.30	1.591	−0.04
**7**	1.62	1.627	−0.41	1.621	−0.08
**8**	1.56	1.558	0.13	1.559	0.06
**9**	1.56	1.558	0.11	1.560	0.00
**10 ***	1.76	1.634	7.14	1.626	7.59
**11**	1.65	1.649	0.05	1.652	−0.14
**12**	1.66	1.659	0.07	1.660	−0.02
**13**	1.97	1.971	−0.06	1.980	−0.48
**14**	2.07	2.054	0.78	2.037	1.58
**15**	1.84	1.844	-0.22	1.862	−1.19
**16 ***	2.41	2.070	14.09	2.094	13.10
**17**	1.79	1.797	−0.41	1.800	−0.53
**18**	1.99	1.987	0.16	1.967	1.16
**19**	1.89	1.893	−0.15	1.902	−0.62
**20**	2.01	2.014	−0.21	2.042	−1.60
**21**	1.98	1.973	0.35	1.962	0.89
**22 ***	2.01	1.917	4.61	1.873	6.82
**23 ***	1.63	1.745	−7.04	1.701	−4.35
**24**	1.78	1.785	−0.28	1.800	−1.10
**25**	1.76	1.769	−0.51	1.738	1.22
**26**	1.78	1.769	0.63	1.770	0.57
**27**	2.35	2.357	−0.29	2.351	−0.03

Figure S1. Plot of the experimental data versus the predicted values obtained from CoMFA model.

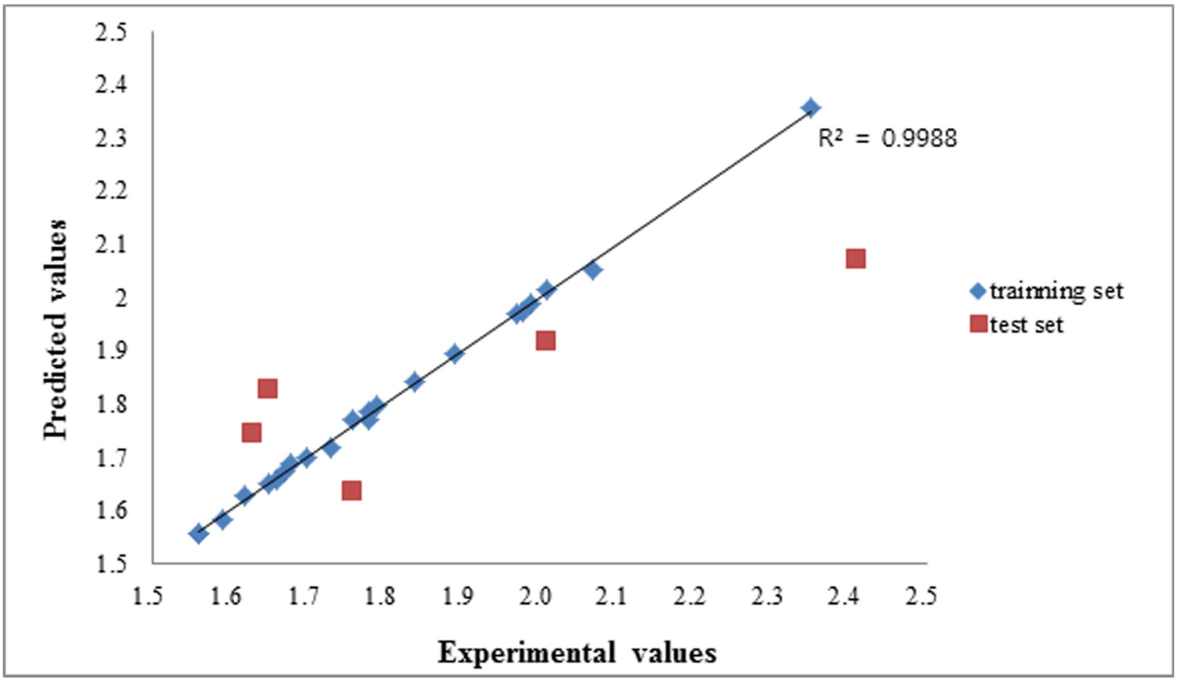

Figure S2. Plot of the experimental data versus the predicted values obtained from CoMSIA model.

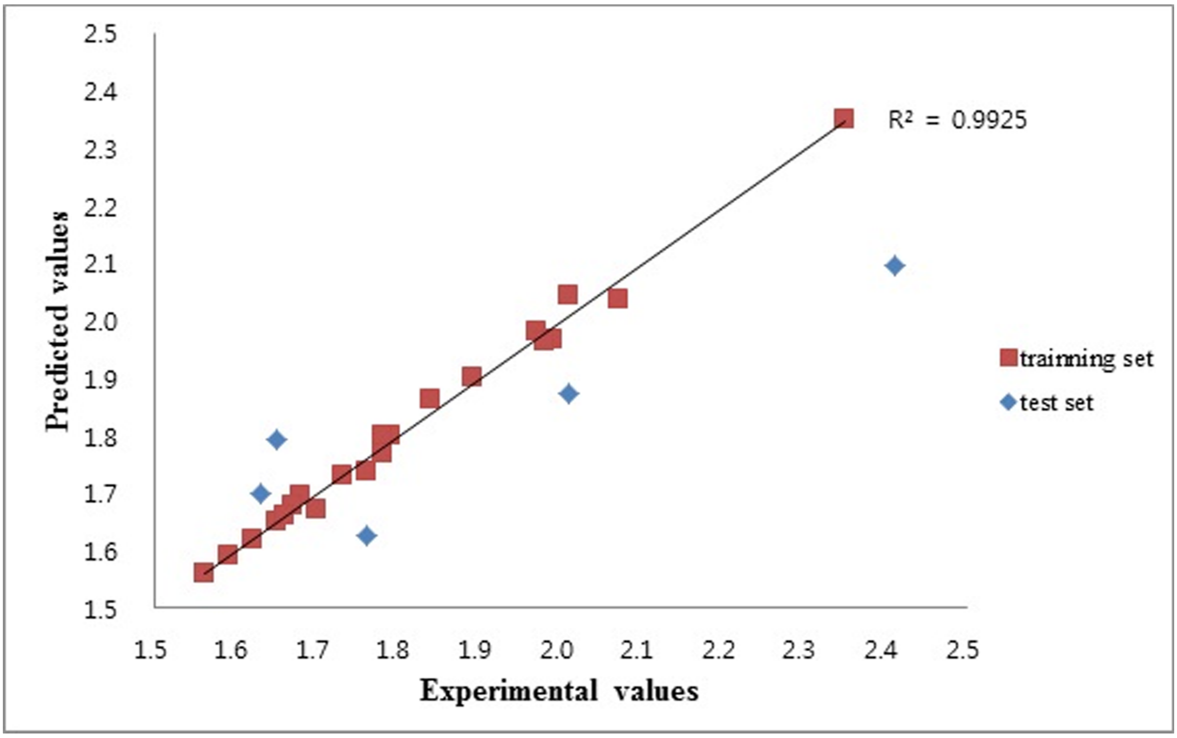

Figure S3. The scaffolds used to obtain the 3D structures of polyphenols **6**–**27**, where the structures were constructed based on the X-ray crystallographic structures.

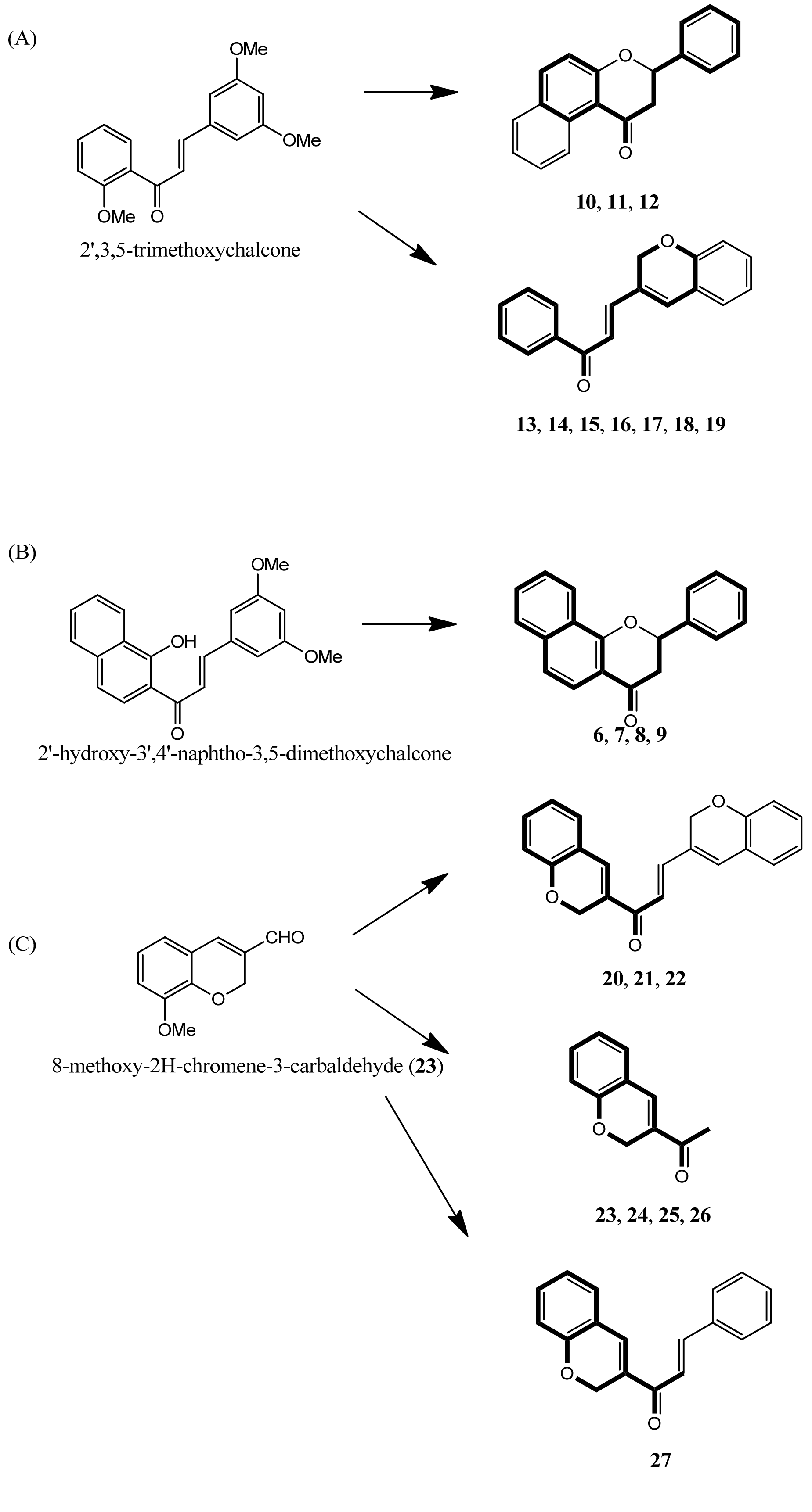

Figure S4. Alignment of training set and test set compounds on polyphenol **9**.

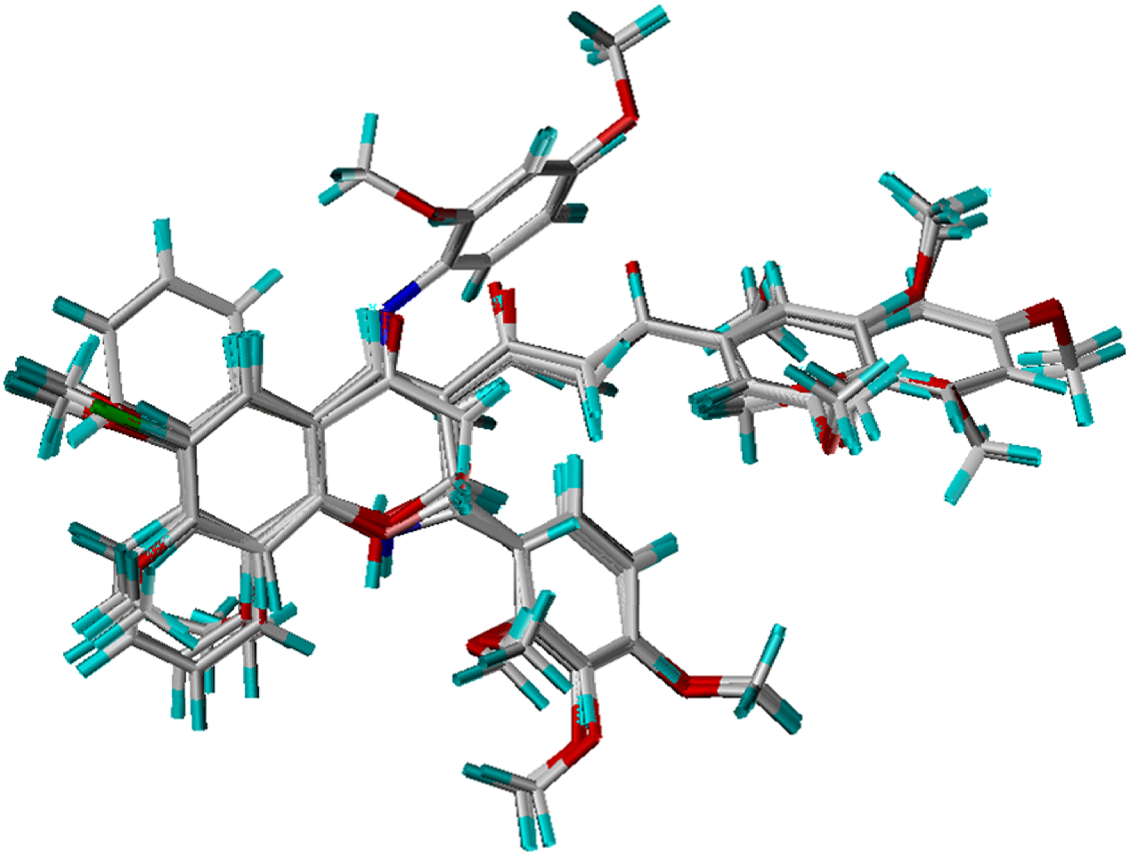

Figure S5. Hirachiral cluster analysis.

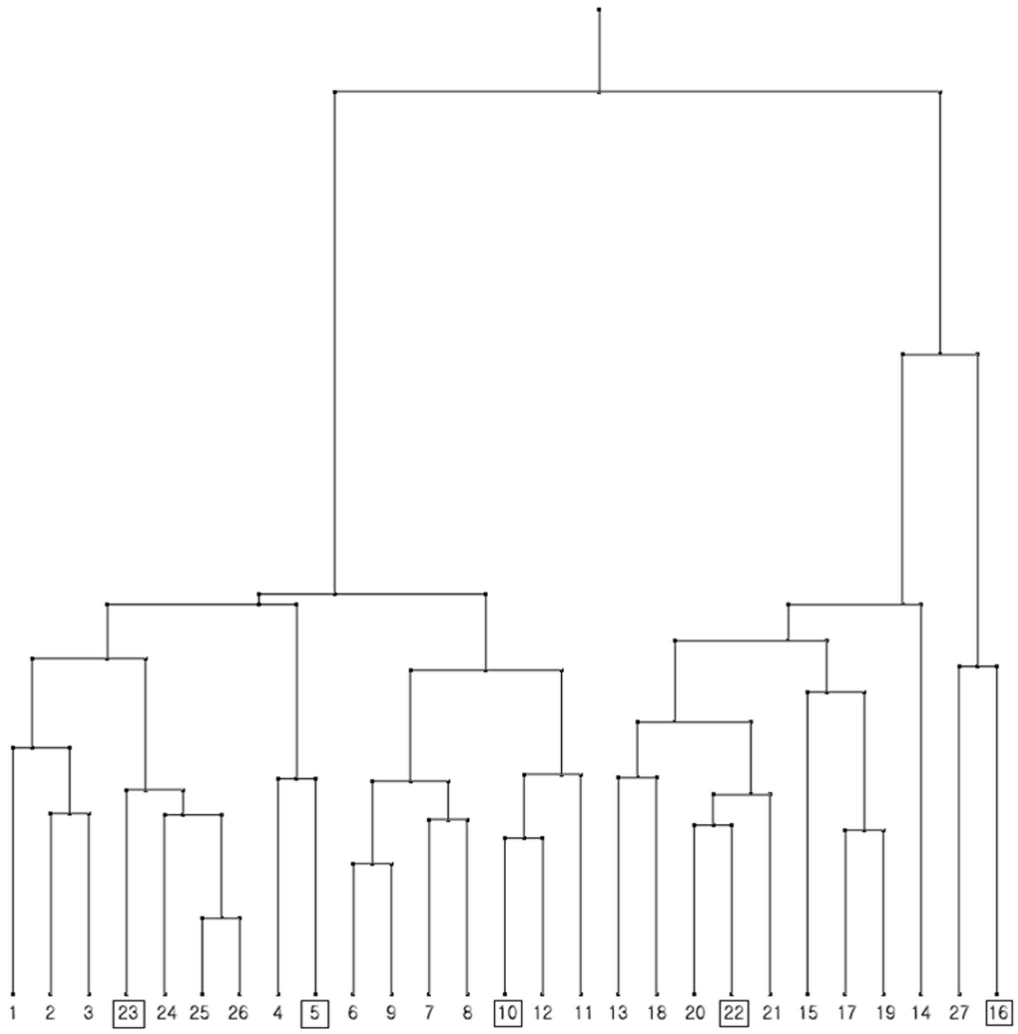

Scheme S1. The procedure for the preparation of polyphenols **6** and **9**.

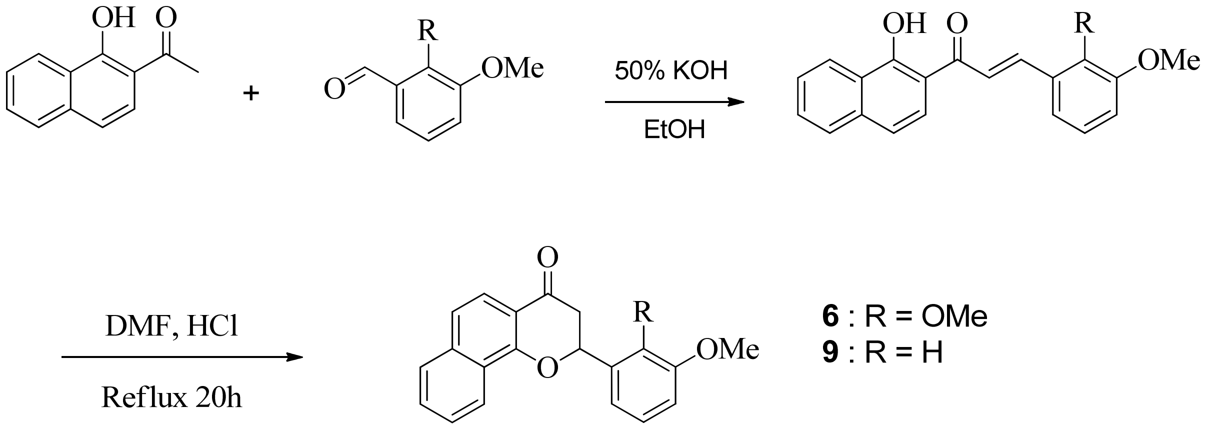

6 : R = OMe
9 : R = H

© 2013 by the authors; licensee MDPI, Basel, Switzerland. This article is an open access article distributed under the terms and conditions of the Creative Commons Attribution license (http://creativecommons.org/licenses/by/3.0/).
